# Co‐creating system‐wide improvement for people with traumatic brain injury across one integrated care system in the United Kingdom to initiate a transformation journey through co‐production

**DOI:** 10.1111/hex.13712

**Published:** 2023-01-30

**Authors:** Kim Manley, Karen Saunders, David Wilkinson, Rafey Faruqui, Mohamed Sakel

**Affiliations:** ^1^ Practice Development and Co Director ImpACT Research Group, Faculty of Medicine and Health Sciences University of East Anglia Norwich UK; ^2^ England Centre for Practice Development, Faculty of Medicine, Health & Social Care Canterbury Christ Church University Canterbury Kent UK; ^3^ Division for the Study of Law, Society and Social Justice, School of Social Policy, Sociology and Social Research University of Kent Canterbury UK; ^4^ Department of Physiotherapy and Rehabilitation Jashore University of Science and Technology Jashore Bangladesh; ^5^ Director of the Division of Human and Social Sciences, School of Psychology University of Kent Canterbury UK; ^6^ Department of Psychiatry Kent and Medway NHS and Social Care Partnership Trust Maidstone UK; ^7^ Division for the Study of Law, Society and Social Justice, Centre for Health Services Studies University of Kent Canterbury UK; ^8^ Director of Neuro‐Rehabilitation Service East Kent Hospitals University NHS Foundation Trust Canterbury UK

**Keywords:** co‐production, neuro‐rehabilitation, person‐centred care, practice development, system and workforce transformation, transdisciplinary, traumatic brain injury

## Abstract

**Background and Objective:**

There is a need for better integration of services across communities and sectors for people living with traumatic brain injury (TBI) to meet their complex needs. Building on insights gained from earlier pilot work, here we report the outcomes of a participatory workshop that sought to better understand the challenges, barriers and opportunities that currently exist within the care pathway for survivors of TBI.

**Methods:**

A diverse range of stakeholders from the acute and rehabilitation care pathway and the health and social care system were invited to participate in a 3‐h workshop. The participants worked in four mixed subgroups using practice development methodology, which promotes person‐centred, inclusive and participatory action.

**Results:**

Thematic analysis identified shared purposes and values that were used to produce a detailed implementation and impact framework for application at both the level of the care interface and the overarching integrated care system. A variety of enablers were identified that related to collective values and behaviours, case management, team leadership and integrated team working, workforce capability, evidence‐based practice and resourcing. The clinical, economic, cultural and social outcomes associated with these enablers were also identified, and included patient safety, independence and well‐being, reduced waiting times, re‐admission rates, staff retention and professional development.

**Conclusion:**

The co‐produced recommendations made within the implementation and impact framework described here provide a means by which the culture and delivery of health and social care services can be better tailored to meet the needs of people living with TBI. We believe that the recommendations will help shape the formation of new services as well as the development of existing ones.

**Patient or Public Contribution:**

Patient and public involvement have been established over a 10‐year history of relationship building through a joint forum and events involving three charities representing people with TBI, carers, family members, clinicians, service users, researchers and commissioners, culminating in a politically supported event that identified concerns about the needs of people following TBI. These relationships formed the foundation for the interactive workshop, the focus of this publication.

## 1 INTRODUCTION

Severe traumatic brain injury (TBI) has a profound impact on people, their carers and families, especially evident when transitioning from hospital to home and the community.^1^ Care is experienced as fragmented, and there is an urgent need for better integration across health and social care and the voluntary sector to enable people with TBI to be at the heart of care.^2^

TBI is a major challenge both globally^3^ and for the UK healthcare system.^4^ As a major cause of long‐term disability, TBI can affect all areas of daily life, reducing the quality of life significantly for both the person and their carers.^1^, ^2^, ^3^ The transition from hospital to home and the community across patient pathways tests the principles of integrated health and social care systems to its limits,^2^ and so getting it right for this group of citizens would provide key lessons for all.

Whilst this challenge affects all societies internationally,^3^ this paper shares early steps towards transforming services across one integrated care system (ICS), Kent and Medway, in England based on a 10‐year history of building stakeholder relationships. Founded on insights about how the care pathway is experienced^2^ and using the key principles associated with co‐production^5^ and practice development (PD),^6^ this service development initiative describes and outlines a specific process that has been used to begin to develop more integrated person‐centred, safe and effective care and services across the health and social care system. Working in partnership with people who have experienced TBI, their families, carers predominantly through key charities, and other stakeholders, the global approach of ‘what matters to you’^7^ underpins the purpose and direction of the transformation required.

### 1.1 The local context

An epidemiological report^8^ published by Kent and Medway Observatory identified that the incidence of TBI has increased for two successive years (2017–2019),^8^ with the total number of in‐patient hospital admissions for TBI increasing from 3645 (2016/17) to 4295 (2018/19). There is no available comparative hospital admission data for this period nationally. The latest regional data for the South East of England for 2019–2020 shows that the rate of finished admission episodes for head injuries has increased by 12% since 2005–2006.^9^

These rates of TBI in Kent and Medway are consistent with European rates. Other key findings from the Observatory report can be found in Box 1.

Box 1. **Findings from the Kent and Medway Observatory report 8**

TBI represents approximately 1% of all admissions (elective and emergency) to hospitals in Kent and Medway.

A higher level of deprivation appears as a risk factor for TBI in Kent and Medway, both in terms of incidence (by initial admission) and for subsequent readmission.

Male sex and older age both appear as risk factors for TBI in Kent and Medway.

West Kent appears to have a significantly higher rate of TBI than the rest of Kent and Medway.

White British people count for the majority of patients with TBI, but the incidence is highest in ethnic minorities in Kent and Medway.

**Figure 1:**
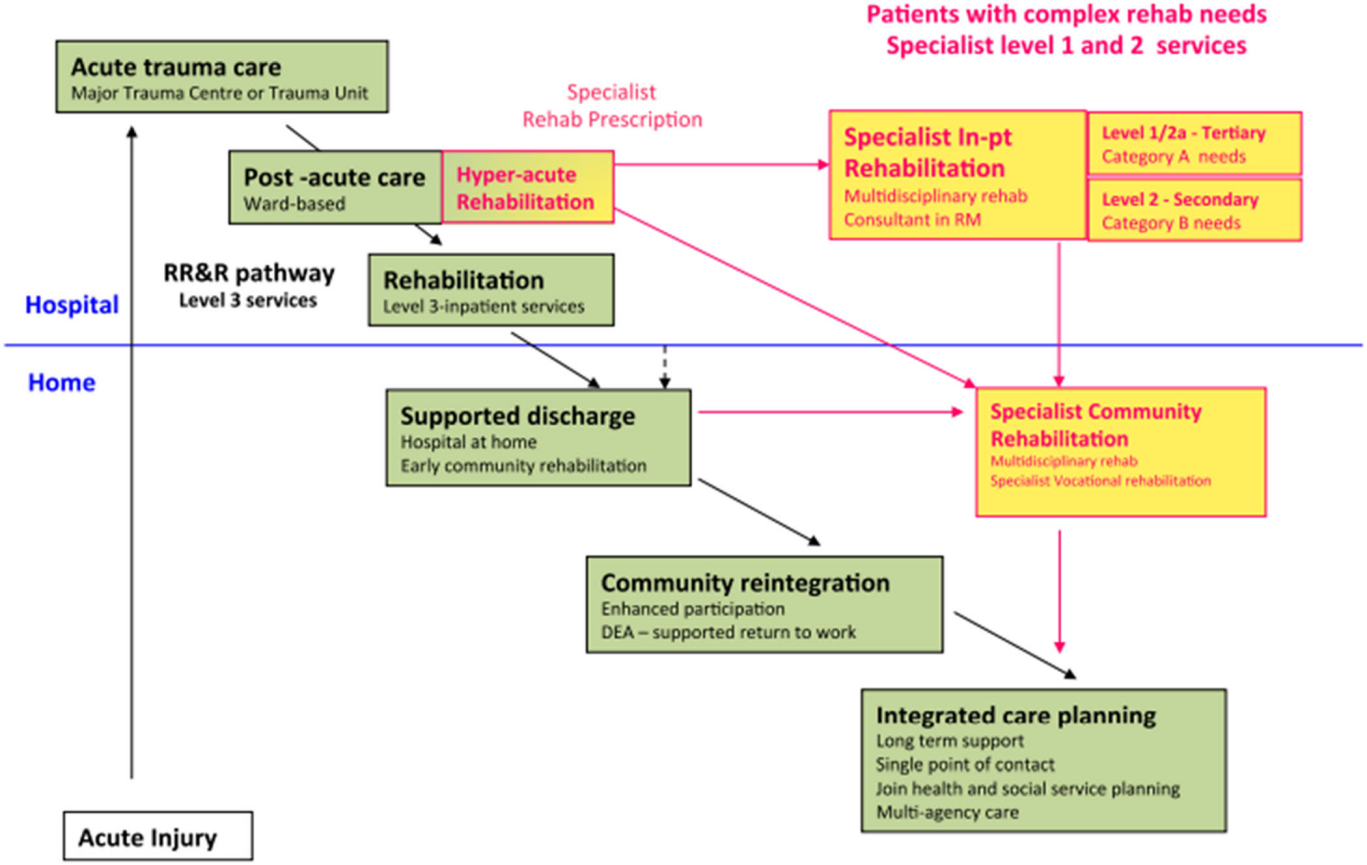
Rehabilitation pathway following trauma as recommended by the British Society of Rehabilitation Medicine.

East Kent has an established in‐patient Neuro‐rehabilitation Service, which provides specialist multi‐disciplinary care to people diagnosed with TBI. Following treatment, individuals are discharged and referred to community services for postdischarge support. However, the postdischarge community pathway (Figure 1) identified in the rehabilitation care standards^10^ lacks definition, and there are limited resources and capacity to meet the ongoing rehabilitation needs of TBI survivors after discharge.

The newly created ICS for Kent and Medway in England has inherited 10 years of development work under the auspices of the Kent Brain Injury Forum (KBIF), a vibrant community of people living with TBI, and their families and carers (experts by experience). These groups have been supported by three charities, all of which have regularly come together to exchange best practices at annual brain injury conferences organized and chaired by the Service Director of the Neuro‐rehabilitation Unit. Key themes emerging from these meetings catalysed an initial research study^2^ to gain insight into the experiences of people diagnosed with severe TBI, who were being discharged home from the local in‐patient service. Insights were also gained from their carers during the first‐month post‐discharge. This study concluded that patients and carers struggled to say what meaningful support had been given for transition into community living. It reported that, following discharge, there were new unanticipated needs that remained unresolved. This study also confirmed that patients and carers require further support in the longer term after the first month postdischarge in the transitional period.

In light of these postdischarge difficulties and the increasing incidence of TBI in Kent and Medway, the Neuro‐rehabilitation Service Director in East Kent decided to convene a consultation with service providers and users at a stakeholder event in the House of Commons in October 2018.^11^ The overarching aims of the event were to (1) raise awareness of the needs of TBI survivors and of the importance of specialist rehabilitation and care services, (2) engage diverse stakeholders across all sectors and (3) expand the East Kent service to include unmet TBI needs in Medway and West Kent (this predated both the COVID‐19 pandemic and the new ICS structures introduced in July 2022). This event comprised 31 attendees, including 6 experts by experience (1 individual living with TBI and 5 family members who were carers). One of the family members was a mother of a TBI patient and an active member of the KBIF charity at that time. Also in attendance were two member of parliament (MP)s: the Chairman of the All‐Party Parliamentary Group on Brain Injury Care service development, and a local MP.

After this launch event, an interactive follow‐up co‐production workshop was organized to explore ways of creating a more effective care pathway that was based on a better understanding of what matters to people who have experienced a TBI, their families and carers. Here we describe the insights and recommendations drawn from that workshop.

### 1.2 Methodology

**Figure 2:**
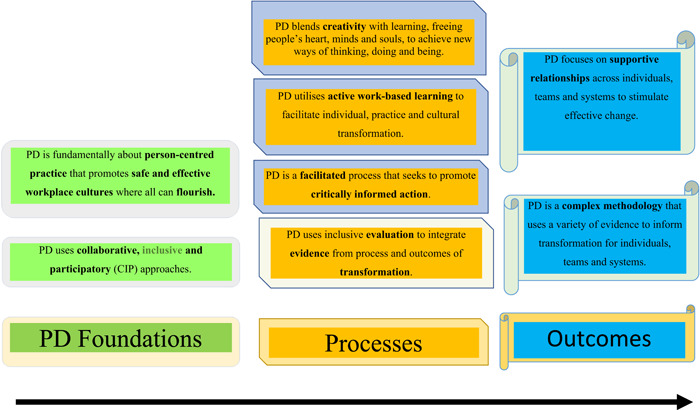
Practice development (PD) principles—a global manifesto^6^.

The interactive workshop adopted a ‘practice development’ methodology, which is detailed in Figure 2.

PD is a useful tool for formulating new innovations and services and is conducted ‘with’ rather than ‘on’ people. In this instance, PD was used to co‐create the purpose, direction, activities and outcomes to inform how TBI services across this ICS should be formulated. PD was selected to guide the initiative because of (1) Its underpinning values—the provision of seamless, person‐centred, safe and effective care across communities with continuity, enabling everyone (providers and recipients of care/services) to thrive and flourish; and (2) Ways of working that are collaborative, inclusive and participative to co‐create outputs with stakeholders and service users enabling active engagement and ownership drawing on the expertise and experiences of all.

PD has much in common with the concept of ‘co‐production’. While there is no single agreed consensual definition of co‐production in the literature, Langley et al.^12^ suggest that ‘co‐production’ can be seen as a way of working with people as ‘knowledge users’^12^ (p. 112) while Kothari et al.^13^ define co‐production as ‘a model of collaborative research that explicitly responds to knowledge user needs in order to produce research findings that are useful, useable and used’ (p. 1). In 2018, the NIHR published guidance on how to co‐produce a research project^14^ which identified key principles and features, whilst acknowledging that there is no ‘one size fits all approach’ (p. 5). The five key principles identified relate to power sharing, inclusivity of perspectives, relationship building, respecting and valuing all participant contributions and gaining benefits from working together. These principles were all integral to the way that the workshop was conducted on the day and served to enhance the PD methodology used.

## 2 METHOD

A wide range of diverse stakeholders was invited to take part in a 3‐h workshop. These were all connected to the East Kent Neuro‐rehabilitation Service, and also included representation from key partner agencies and charities across the acute and rehabilitation care pathway and system. Three key charities (KBIF, Kent MS Therapy Centre and Headway) attended along with a carer representative, expert clinicians, managers and a neuro‐rehabilitation commissioner, all of whom had been unable to participate in previous meetings. The workshop was co‐facilitated by the Neuro‐rehabilitation Service Director and a Professor in Practice Development and was coordinated and supported by a core stakeholder group. Twenty‐five individuals participated overall (Table 1).

**Table 1:** Stakeholder representation from across Kent and Medway in the workshop.

Total number of participants	Clinicians	Family carers	Secondary care managers	Charity representatives	Commissioners	Researchers	Legal representative solicitor
25	10	1	7	3	2	1	1

The event was intentionally organized on a central hospital site to enable those hospital clinicians to participate who had been unable to leave the hospital to attend the initial launch event in London. The PD processes used in the workshop are summarized in Table 2.

**Table 2:** Practice development methods, workshop processes and outputs.

Method/process (P)	Detail	Outputs and outcomes/data
P1. Advanced briefing information to prepare participants	Draft ways of working	Agreed ways of working in the workshop to enable a safe environment
	Preparatory questions:	
		Informed:
	(1) What is your ONE most important passion when caring for/providing services to people with TBI requiring rehabilitation? (2) What do you believe to be the ULTIMATE PURPOSE of care and services for people with TBI requiring rehabilitation across Kent and Medway?	
		(1) Identifying what matters to participants (informed P2) (2) Values clarification (informed P3)
P2. Identifying what experiences matter to participants when caring for/experiencing services for people following brain injury requiring rehabilitation?	Participants asked to join one of four groups linked to what mattered to them and their experiences of caring for people with TBI requiring rehabilitation:	Themes (1st level analysis) collaboratively developed by participants in each of the four groups, informed the implementation and impact framework
	(1) Successes, (2) Challenges, (3) Obstacles (4) Opportunities	
	Each group identified three bullet points capturing shared experiences	
P3. Values clarification^37^	Four workshop groups worked in parallel to verbally buzz responses in relation to the following stems, whilst a volunteer note taker captured points on separate post‐its:	Regarding stems 1–2
		Each group developed a statement of ultimate purpose and agreed four ways of achieving the purpose (stems 1–2) which were shared with other groups and collated electronically to inform purpose (1st level analysis)
	(1) I/We believe the *ultimate purpose* of services for people with TBI needing rehabilitation is. (2) I/We believe this purpose can be *achieved by* the following *four bullet* points. (3) I/We believe *enablers* to achieving the purpose are. (4) I/We believe *effective ways of working across the system* in Kent & Medway that support the purpose include. (5) I/We believe the *FIVE key interventions/capabilities* required by the person with brain injury needing rehabilitation are. (6) I/We believe our *success indicators* would include. (7) *Other values and beliefs* I/we hold to be important are.	
		All post‐it notes from all group's responses for stems 3–7 were placed on a flipchart dedicated to each stem to be used for collaborative theming (P4)
P4. Collaborative theming of post its on each flipchart undertaken by different groups thematic analysis	1st level thematic analysis. This involved each group working with the data for single stems from all four groups:	Collaborative themes
	(1) Reviewing post‐it content, (2) Clustering post‐its into themes, clarifying meaning if needed, (3) Using the language on the post‐its to compile a theme label	
P5. Implementation and impact framework informed by concept analysis frameworks	Participant Groups decided where their themes tentatively sit on the implementation and impact framework (2nd level analysis), that is:	Themes from collaborative analysis in groups are placed on the implementation and impact framework
	(1) An enabler (2) An attribute of the system/service (3) Consequence, output, outcome	
P6. Unfolding story	A creative approach to help people ‘think outside the box’ about the service, its vision and direction	This was collated after the workshop for later development
P7. Evaluation	Participant groups invited to identify the words that describe their experience of the workshop. Then use these words to generate a three‐line poem (Haiku) to reflect this	Shared with other participant groups to capture the evaluative experience of being involved

Process 1 commenced before the workshop to enable participants to take an informed decision to participate, and to know both what to expect and the preparation required. What mattered to participants in relation to services for people with TBI would be an important starting point guided by participants' experiences, values and expertise. Participants needed to know that their voice mattered, and this would be enabled through agreed ground rules to enable openness, honesty, creativity and learning for shared mutual understanding. Outputs from the workshop would be synthesized from group activity collaboratively and would not be linked to any one individual or organization. Whilst the workshop was under the umbrella of collaborative service improvement rather than research, the need for a safe psychological environment was essential to enable everyone's voice to be heard. Anonymity for participants was protected in relation to the data arising, which was group‐level data, not individual data.

Process 2 aimed to distil what mattered to participants about their experiences of receiving or providing TBI services and would help identify learning from past successes, challenges, obstacles and opportunities paving the way for a formal values clarification exercise. Participants were self‐selected to a chosen group focusing on either successes, challenges, obstacles or opportunities and distilled 3‐4 common themes from their individual experiences.

Process 3 involved participants working together in premixed stakeholder groups to complete a values clarification exercise to identify the shared purpose and ways of working, enablers, key system, and service features, and expected outcomes. Small group work with mixed stakeholder participants was intended to endorse a safe space for different perspectives, with everyone's voice expected to be heard. Everyone's contribution was verbally shared and captured on ‘post‐it’ notes.

Collaborative analysis (Process 4) achieved by clustering the post‐it notes from the previous activities was shared across the four workshop groups. Participants were asked to identify common themes, use the language of participants to describe themes, and clarify meaning where this was not clear rather than making their own interpretations.

**Figure 3:**
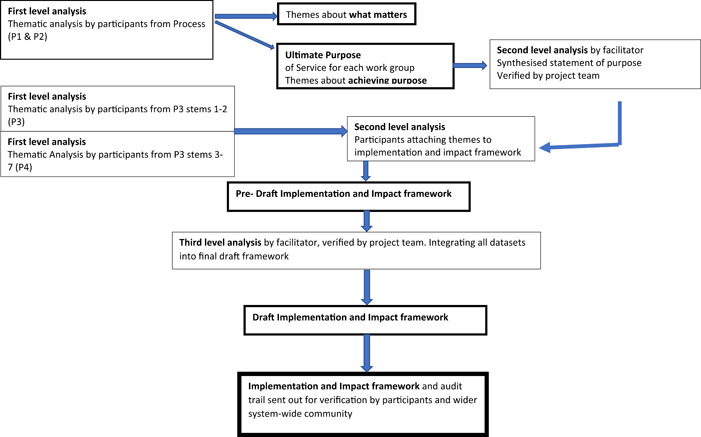
Data analysis flow chart leading to the synthesis of the implementation and impact framework from workshop processes (P).

The themes generated collectively were then allocated to an implementation and impact framework by participants (Process 5). This framework comprised three parts—the enablers required to support system transformation and improved services for people with TBI; the activities or attributes that would characterize what the system and the pathway would be doing when caring for people in Kent and Medway with TBI, and lastly, the anticipated impact that would guide the evaluation of the system's effectiveness. Figure 3 describes the analysis process to explain how the results were generated.

Process 6 involved a creative exercise to help participants to think ‘outside the box’ and to envisage a new future for the service, which could be developed in future sessions.

The final workforce process (P7) focused on creative evaluation, an important part of PD to capture both collaborative learning and how the workshop was experienced in relation to the collaborative, inclusive and participative space intended.

## 3 RESULTS

The interactive processes between individuals and groups within this workshop, which can be described as co‐production (Langley et al.^12^), enabled participants to identify and share core priorities and enabled the development of an implementation and impact framework to guide integrated services for people with TBI at two levels:

(1) at the micro level—which refers to the frontline teams interfacing directly with people living with TBI across the care pathway;
(2) at the macro level, which identifies what the ICS needs to do to support an improved care pathway.

The key values and priorities that were identified included:

(1) Restoring the quality of life after brain injury with stories that tell this;
(2) Involving families to ensure support is provided for all;
(3) Collective objective‐setting;
(4) Integrated services across all parts of the pathway;
(5) Funding to achieve values and meet demand.

The shared purpose that emerged from the four mixed working groups to guide the transformation of services (see Box 2) was:

*To enable people to optimise their rehabilitation potential and return to a meaningful life, maximising their quality, participation and independence*

Box 2: **Synthesizing the ultimate purpose for care for people with traumatic brain injury from the four workshops group purposes**

**Table:** 

Synthesized shared purpose	Groups 1–4 agreed ultimate purposes × 4
Ultimate purpose is: *To enable people to optimize their rehab potential and return to a meaningful life, maximizing their quality, participation and independence*	To enable people to achieve their optimal rehab potential and long‐term quality of life
	To maximize access and opportunity to enable the patient to achieve optimum function and participation and support families and wider services in this journey
	Reducing dependence and maximizing quality of life
	To return to a meaningful life

Two interrelated implementation and impact frameworks were developed from themes generated at the workshop for the purpose of identifying the enablers, activities and indicators for evaluating impact with a specific focus on (1) individuals with TBI and their direct care teams, (2) the broader ICS that supports the TBI pathway.

The implementation and impact framework summarized in Table 3 focuses on what participants feel is needed by people with TBI, their carers and families at the micro‐system level and across the pathway. The framework identifies the key activities that the service needs to focus on for those directly experiencing it, namely: engaging people actively about what matters in partnership with those providing it; delivering ongoing holistic care and support and flexible patient pathways that include active follow‐up. The enablers include collective values and behaviours that support people with the activities identified from the perspective of both those providing and experiencing care; case management, team leadership with the integrated team working; workforce capabilities and evidence‐based practice across the pathway; time and support for staff to be responsive. The emergent impact is expected to improve patient participation and safety, as well as the wider quality of life and independence.

**Table 3:** An implementation and impact framework for people following traumatic brain injury at the microsystems level and the teams providing direct care.

Enablers	Activities/attributes	Outcomes/Impact
*Collective values and behaviours*	*Engaging actively with people and their families are engaged*, with teams working in partnership to: (1) Identify what matters to them (2) Optimize rehabilitation potential for returning to a meaningful life *Providing on‐going holistic care and support* to patients and families through the multiprofessional team to: (1) Develop a person‐centred care plan with follow‐up (2) Ensure safety (3) Manage emergencies, for example, seizures, alert cards, and so forth (4) Provide advice, guidance including benefits, rights *Flexible pathways* to enable: (1) The person and their family to know what to expect across the patient journey (2) Home visits with active support by therapists to integrate person back into home life (3) Follow‐up and revisiting over time	*Patient and family impact process outcomes*
(1) Compassion and care, (2) Respect, dignity and hope (3) Staff, patients and families are motivated, committed, inspired, driven and actively listen to each other *Case management*		
		(1) Patients and relatives more informed (2) Increased participation (3) Safety maintained *Longer‐term outcomes*
(1) Case management to enable continuity Team leadership and integrated team working		
		(1) Achieved optimal rehab potential (2) Increased independence (3) Increased quality of life (4) Restored long‐term Quality of life/Returned to a meaningful life (5) Increased return to work rates *Patient, family and staff experience*
(1) Effective leadership integrating team expertise and commitment to maintain high quality (2) Shared vision and common goals, embracing different perspectives backed by clear strategy, policy and effective leadership (3) Integrated team working and joined up thinking across the pathway and patient journey with mutual synergy and joined up working (i) Staff work across boundaries *Workforce capabilities and evidence‐based practice*		
		(1) Improved experiences (2) Patient and family satisfaction (3) Patient feedback benchmarked (4) Less complaints (5) People feeling valued (6) Staff recruitment and retention
(1) A well‐educated, skilled workforce, up to date with current evidence‐based practise within neuro‐rehabilitation (2) Multidisciplinary capabilities framework across the pathway *Resources: Time and support to be responsive*		
(1) Adequate resources, for example, staff provided with time and support for patients when they find themselves plugging a hole/if no service elsewhere *System enablers—see systems framework* (see Table 4)		

The system‐level enablers and activities required to support the pathway and the micro‐system level are presented in Table 4. Key enablers include systems leadership to drive integration, workforce development, a learning culture that supports education and research and stakeholder commissioning for a joined‐up pathway.

**Table 4:** A system‐wide implementation and impact framework for supporting people and workforce teams caring for people following traumatic brain injury across place.

Enablers	Activities/attributes	Outcomes/impact
*Systems leadership that drives integration* (1) Effective leadership that integrates team experts with ‘joined‐up’ working across the whole pathway and patient journey, for example, specialist services input to include neuropsychiatry and neurobehavioral/neuropsychological expertise (2) Multidisciplinary interagency and interprofessional working, sharing and supporting across all health and social care sectors *Workforce development and resources* (1) Workforce development through a multiprofessional career and capability framework (2) Resources: people with skills, time, equipment, facilities, for example, day hospital; (3) Funding to pursue innovation and technology *A learning culture that supports education and research* (1) A forum for learning that is resourced (2) Education opportunities for all: individuals, partners and agencies in care (3) A diverse research programme, comprising multiple groups and integrated into the pathway to improve both short and long‐term patient and family outcome. These groups will also be able to make an educational contribution *Stakeholder commissioning for a joined‐up pathway with*: (1) Equity of access to services (2) Integrated communication system (3) Infrastructure for population health management (data) *Funding* (1) Opportunities for establishing long‐term funding across sectors and expansion of the service county‐wide	Creating engagement opportunities for people with emphasis on care in the community Enabling active participation in education and learning by staff, patients and the wider community Championing and celebrating people, continuous improvement and innovations, for example, improving options for appropriate supportive living in the community Using population health data for planning Broader engagement with society	*Outcome measures of performance, quality and clinical outcomes* (1) Impact indicators for person, families and team (see Table 3) *System process indicators* (1) Reduced length of stay (2) Reduced admissions (3) Re‐admission rates (4) Reduced waiting list to access services/clinics (5) Reduced referrals to mental health, criminal justice, alcohol and drug abuse (6) Early intervention (7) Increased synergy across health and social care (8) Removal of systems barriers *Learning culture* (1) Lifelong learning for people and staff *Population* (1) BAME population integration and pathway monitoring *Society impact* (1) Involvement of Brain Injury volunteer organizations (2) Normalizing, campaigning, legislation

An example of how themes were generated to populate Tables 3 and 4 is provided in Box 3, where the enabler of ‘effective systems leadership’ identified by one workgroup is illustrated from the five individual ‘post‐it’ notes that comprised the theme.

Box 3: **An example of first‐level data analysis from group written post‐it notes to generate a theme title for one enabler at the systems level**

**Table:** 

Theme identified by workshop group working with enablers	Individual post‐it notes written by individuals comprising the theme
Effective leadership that integrates team experts and joined up working across whole pathway and patient journey	Team, good leadership and followship without silos Integrated team experts across the whole pathway Integrated team efforts across the whole pathway Mutual Synergy and joined up working Support across system barriers—health and social services

Key activities that the ICS needs to embrace include: creating social engagement opportunities for professional stakeholders; promoting active participation in ‘learning for all’ events; championing and celebrating continuous improvement and innovation and using population data for planning. The outcomes and impact are those most relevant to people with TBI and their families, however, staff outcomes, such as well‐being and job satisfaction, were also recognized as influential on the quality of care experienced.

### 3.1 Evaluation of the workshops

Evaluation is vital not just to ascertain whether outcomes were achieved and how they inform continuous learning and improvement, but also to establish whether collaboration, inclusion and participation produced a genuine co‐productive experience.^6^ In keeping with the principles of PD to use inclusive evaluation approaches that endorse co‐production that focus on what matters to people, PD also blends creativity with learning to free peoples' hearts, minds and souls to achieve new ways of thinking, doing and being (see Figure 2). Participants on their tables were encouraged to capture words that best described their experience of the workshop. Participants were then given guidance about how to structure a Zen poem, termed a Haiku, with its first line of five syllables, the second line of seven syllables and the third line of five syllables and were invited to work with their chosen words to develop a collaborative poem that expressed their combined experience. The resulting expressions are shared with the other groups, and are frequently experienced by participants as powerful, uplifting and energizing and genuinely convey their lived experience. In other contexts, creativity can also include the use of nature, mandalas, artwork, images and metaphors to express an individual or group journey and learning. The evaluation (Box 4) demonstrated a positive, collaborative and empowering experience for participants that felt friendly, respectful, stimulating and thought‐provoking, and reflected achievement of the top rungs of the co‐production and co‐design ladder—that is, ‘doing within an equal and reciprocal partnership’.^15^

Box 4: **Evaluation poems (Haikus) to convey the experience of each work group**

**Table:** 

‘We feel empowered Stimulated, united Curious—next steps?’	‘Creative, friendly Mutually respectful Inspirational’
‘Collaborative Thought‐provoking & well‐led Positivity’	‘Expansive interest Friendly collaboration Welcoming, Mixing’

## 4 DISCUSSION

The shared direction and purpose co‐created from the workshop were to find better ways to ‘optimise rehabilitation potential and return to a meaningful life, maximizing their quality, participation and independence’.

Developing a shared purpose is the starting point when embarking on a journey of collaborative transformation, major change and sustainable improvement,^16^ but its achievement requires shared meaning at all levels of the system and genuine engagement with people and their families about what matters to them.^7^

Our workshop highlighted several core principles of care pathway improvement:

(1) Focus on powerful emotions for good^17^;
(2) Inform conversations by case managers and professionals about personal goals, needs, care planning and continuity of care, identified as lacking in previous research^1^, ^2^;
(3) Measure what matters and what is valued^18^ rather than what is mandated^19^ and
(4) Inform continuous learning and improvement.^18^, ^20^

Facilitating and supporting emotional work with people is a prerequisite for compassionate person‐centred care, which in turn requires team members to have emotional intelligence, and access to the emotional support necessary for sustaining staff wellbeing.^21^ A body of powerful stories can convey the real impact to inform learning, improvement and research supporting transformation by using evaluation approaches that focus on what works or does not work, and for whom in what contexts.^22^

There is a need for improved continuity of care across pathways with flexible follow‐up and access to support contacts, and better communication.^1^, ^2^ Case managers would be pivotal to enable continuity and improvement of care by ensuring that the patient and carer are at the centre of service provision.^2^ Support with meeting the complex care needs of people with TBI is required not just for carers and families, but also by general practice, and community care partners involved in care management across the system.^2^

The UK Brain Injury charity, Headway, defines a case manager as being ‘responsible for overseeing and managing the overall care of the person with a brain injury. They will prepare an individually tailored care plan or treatment programme for and with each client, which is designed to meet the person's specific health, social and emotional needs’.^23^

Whilst Headway recognizes that case managers come from different professional backgrounds, they assert that in the United Kingdom, they are only usually available through private referrals and interim compensation payments. By contrast, in Australia, a community‐based case management system exists^24^ providing consistent direct, holistic, client‐centred services, with decision‐making directed equally by staff and clients. That said, the healthcare system in Australia is funded differently and is based on healthcare insurance paid by Australian taxpayers. The challenge across the United Kingdom would be to consider how an ICS could potentially accommodate and support the introduction of such a role.

Mindful of Berwick's^18^ plea in the context of the third era of healthcare, ‘to measure only what matters, and mainly for learning’ (p. 1239), demonstrating positive impact and progress towards achieving person‐ and family‐centred goals, therefore, require indicators and measures that reflect the needs of people experiencing TBI.

Whilst broad performance indicators linked to experiences of care are routinely collected for services in England, such as the Friends and Family Test and patient satisfaction surveys, little effort is put into using this data to support service improvement.^19^ The professional skills training required to help teams and systems take forward any insights gained from these data are also often missing, as are the governance systems that integrate learning with improvement and research at the meso and macro system levels.^22^

There is a large range of standardized outcome measure indicator scales which are currently used by medical and health care professionals within the clinical practice to establish individual baseline performance and measure change over time, for example, the Glasgow Coma Scale^25^ and Modified Ashworth Scale.^26^ These measures are scientific instruments with associated psychometric properties (validity, reliability and responsiveness), which are used to support and guide treatment interventions and also to enable individuals with TBI and their families to better understand associated change and progress, which in turn can serve to support and encourage motivation and engagement in the recovery process. Given that the majority of outcome measures have been developed and designed by clinicians in partnership with academics, the current study underlines the need to co‐produce additional outcome measures to ensure that the needs of people with TBI (and their carers) are properly assessed.

Together with the framework articulated in Tables 3 and 4, these core principles highlight action priorities at the care interface that:

(1) Use and embed emotional touchpoints^17^ with other measures that support learning, enable improvement and celebration, as well as identify the strategies that work, using co‐production approaches;
(2) Consider how people living with TBI and their families can be potentially supported to contribute to improving the care pathway for people post‐TBI in the community;
(3) Consider how people living with TBI and their families can help co‐design relevant outcome measures for use across the care pathway.

Embedding what matters at the microsystem level through using tools derived from experience‐based design, such as emotional touchpoints, provides deep emotional insights when used in everyday practice.^17^ Emotional support has been identified as a key unmet need for both people with TBI and their carers^2^ and this tool can be tailored to people following TBI, their carers and families to explore both positive and negative emotions associated with unmet needs across pathways as well as successes.^17^ Emotional touch points focus on emotional challenges related to different points across the pathway experienced by people as they cope with a disability, enabling both catharsis and a focus on the emotional support required.^1^, ^2^ Positive emotions can also be shared with those at earlier stages in their rehabilitation journey to inspire hope and share strategies that work.

Beyond the care interface, the workshop also concluded that the underpinning health and social care system needs to support frontline teams through the professional development and sustenance of high‐quality multidisciplinary professional teams with capabilities wrapped around individual citizens rather than the professions. This is quite a challenge for the NHS, which is founded and based on individual professions which are regulated by independent regulatory organizations. Legislative barriers exist that specifically define the scope of practice within which a medical and healthcare professional can legitimately and professionally work, so attempting to change roles or practice would require allied legal change. The concept of ‘trans‐disciplinarity’, often used to describe professionals working across traditional boundaries, is poorly defined in research, although Van Bewer^27^ asserts that it involves ‘sharing of knowledge, skills and decision‐making with a focus on real‐world problems’ (p. 346). Given the importance of the patient and family within healthcare, we suggest that the transdisciplinary healthcare team should include individual patients and related nonprofessional stakeholders.

This area of action should endeavour to:

(1) Appoint systems leaders with the prerequisite skill set to ensure a joined‐up approach across ICS and TBI pathways;
(2) Develop a multiprofessional capabilities and career framework (including case management) around each individual's TBI journey;
(3) Create further opportunities for genuine engagement approaches with people and communities through co‐production;
(4) Enable the co‐evaluation of person‐centred, safe and effective resources and services by creating a learning culture across the system at every level, inclusive of all.

System levers and enablers required to support pathways for people with TBI include systems leadership^28^ and workforce development,^29^ a place‐based learning culture^30^ and stakeholder commissioning with indicators of system effectiveness that enable resources (people and infrastructure) to be used for optimal effect across communities.

Workforce transformation is essential for supporting integration across the system, and three enablers have been recognized, namely (1) Systems leaders with the skills to break down barriers and organizational silos; (2) Facilitation expertise to draw on the workplace as the main resource for learning, developing and improving and (3) Recognition of the need to wrap capabilities around the person, citizens and communities.^28^, ^30^

Problems with vertical integration across pathways have been identified as detrimental in the first months at home, whilst case management across the pathway for people and their carers, together with quality team leadership, can positively address this for people experiencing TBI.

Workforce enablers at the systems level include the need to grow interprofessional capabilities tailored to the person with TBI and their carers, recognizing the unique expertise that emerges from collective leadership and working.^31^ This kind of approach would place the ability to collaborate and share best practices at the heart of staff recruitment and development. Developing the right staff capabilities for a quality integrated service where learning is at the heart of the system is a priority.

Developing people‐centred learning cultures at every level of the system is another essential enabler^30^ and, as intimated, relies on forming successful partnerships with people experiencing TBI to ensure effective ongoing service evaluation and innovation.^30^ These partnerships will help move from a deficit model focused on learning from safety incidents towards one that values everyone's contributions in relation to what matters. This involves growing carers as facilitators of change^31^ and increasing the availability of relevant placements and career progression opportunities to develop the practice of health and social care professionals.^30^ These learning cultures require skilled facilitators who can foster an approach to learning that recognizes the financial uncertainty and ambiguity within which the cultures must nevertheless flourish.^6^, ^29^, ^30^, ^32^, ^33^ We suggest that ICSs will know if their services are right for people experiencing TBI if the co‐produced individual outcomes are met and system‐level indicators show reduced lengths of stay, admissions and re‐admission rates, referrals to mental health, criminal justice and substance abuse support services. Getting this right across the system would not only have a positive impact on people with TBI, their carers and families but also on communities and society more generally.

Beyond these recommendations, we suggest that it would also be useful to gain knowledge of what kinds of physical and mental health issues are experienced by people living with TBI in the much longer term. Insight into the nature of health needs experienced chronically, as opposed to soon after discharge, could further inform the framework proposed here (see Brett and colleagues^34^, ^35^, ^36^).

### 4.1 Strengths and limitations

We acknowledge that the implementation of the current framework and sustenance of allied values will depend on multiple factors. The NHS is a complex system within which numerous teams, services and individuals are working under constantly changing conditions, organizational climates and political influences. Together these affect the extent to which a front‐line team can work in partnership with people post‐TBI. The COVID‐19 pandemic has changed work environments and practises and has especially affected the NHS work environment. We are also conscious that individuals who have survived TBI are a vulnerable group who may well have been impacted more negatively during the pandemic and lockdown periods. It is fortuitous that this workshop event took place prepandemic, which enabled a large group of stakeholders to take part without any undesirable health risks. We do note, however, that the workshop would perhaps have been enhanced by even greater citizen involvement. To reiterate a key point from above, we also understand that one learning outcome from the pandemic has been that there is a need to work in much greater partnership with people across the system and build collaborative working relationships. The introduction of ICSs across England provides a template through which to achieve this.

## 5 CONCLUSION

The recommendations made here formulated through a process of co‐production, provide an initial framework by which the experiences of people living with TBI can be improved via system‐wide transformation. Co‐produced improvements are needed postdischarge at both the micro‐level across pathways (to improve the experience and contribution of people with TBI at the care interface) and systems‐level (to improve staff culture, recruitment and progression and increase multidisciplinary expertise that is joined‐up). More broadly, the co‐produced framework model that has emerged could offer a template for other neurological rehabilitation services that are likewise in need of reform.

## CONFLICT OF INTEREST STATEMENT

The authors declare no conflict of interest.

## Data Availability

The anonymized group‐level data collected is not publicly available as this was undertaken as a collaborative service improvement initiative where participation would be protected safely by anonymity and privacy.
